# Soft Tissue Safety Assessment of Pulsed Solid‐State Thulium:YAG Versus Holmium:YAG Lasers: A Quantitative Porcine Model Simulation

**DOI:** 10.1002/lsm.70173

**Published:** 2026-07-15

**Authors:** Dennis Buente, Rodrigo Suarez‐Ibarrola, Shane O'Sullivan, Arkadiusz Miernik, Ralf Petzold

**Affiliations:** ^1^ Department of Urology Faculty of Medicine, University of Freiburg ‐ Medical Centre Freiburg Baden‐Württemberg Germany

**Keywords:** laser parameter, laser pulse power, lithotripsy, pulsed thulium laser, retrograde intrarenal surgery, soft tissue damage

## Abstract

**Objectives:**

Laser lithotripsy carries an inevitable risk of collateral tissue injury. This study compared the safety profiles of pulsed Holmium:YAG and Thulium:YAG lasers by examining their effects on soft tissue under laser settings relevant to lithotripsy, using ex vivo porcine kidney models.

**Methods:**

Direct laser impacts were applied to porcine renal parenchyma and pelvis at constant fiber movements and clinical laser settings (0.2–3.5 J, 10–40 Hz, 2–40 W), with high‐power extensions for the Thulium:YAG laser up to 80 W/200 Hz. From 560 histological sections, coagulation and cutting depths were measured and clustered.

**Results:**

At equivalent power, the Thulium:YAG laser operated with longer pulses than Holmium:YAG, reducing pulse energy per time (pulse power ≤ 3362 vs. ≤ 7777 W). Holmium:YAG laser produced deeper lacerations up to 9.3 mm in renal parenchyma, compared to 5.0 mm with the Thulium:YAG laser. In renal pelvis, maximal cut depth was 3.6 mm with Holmium:YAG—twice as that observed with the Thulium:YAG. While pulse power was a primary determinant for soft tissue damage, high frequencies (100–200 Hz) did not elevate it. Cluster analysis identified laser‐specific damage profiles: Thulium:YAG favored coagulative damage at limited depths, whereas Holmium:YAG induced deep lacerations with limited coagulation and tissue detachment.

**Conclusion:**

Soft tissue safety and complication risk depend on laser parameters and choice of technology. Reduced pulse powers maximize safety, instead pulse frequency seems safe to elevate for improved efficiency. At power levels beyond 20 W, Thulium:YAG benefited from longer pulses showing safer profiles, with shallower penetration and more reliable coagulation compared to Holmium:YAG.

## 1 Introduction

Retrograde intrarenal surgery (RIRS) with laser lithotripsy carries a risk of collateral tissue injury [1]. Superficial lesions typically heal without long‐term consequences, however, deeper damage may result in leakage of irrigation fluid and the formation of urinomas, potentially leading to scarring of the ureter and peri‐ureteral tissue [2, 3, 4]. In the long‐term, this can impair urine flow and increase the risk of hydronephrosis, promoting recurrent stone formation, acute renal colic, and infection. In severe cases, urosepsis or death may occur [5, 6]. Given that the physiological thickness of the ureteral wall and fibrous lining of renal pelvis is approximately 1 mm [7, 8], the recent developments of high‐energy pulses (up to 6 J) pose a considerable safety challenge [9]. Therefore, minimizing laser‐related injury is essential.

Holmium:Yttrium–Aluminum–Garnet (Ho:YAG) laser lithotripsy has established itself as the gold standard in endoscopic stone treatment, achieving high stone‐free rates postoperatively [10]. Laser systems with powers of up to 150 W [9, 11], along with advanced pulse modulation technologies such as Moses Technology [12], have been introduced. Nevertheless, Ho:YAG is only one of several contenders in the field of laser lithotripsy. Fried et al. have investigated diode‐pumped thulium fiber lasers (TFL), reporting promising outcomes [13, 14, 15]. Clinical benefits of TFL include higher stone ablation rates, smaller fiber diameters which increase fluence at same pulse energies, and reduced retropulsion compared to Ho:YAG [16]. The most recent addition to the field is the pulsed solid‐state Ho:YAG laser (p‐Tm:YAG), which is further examined in this study.

Laser‐induced tissue damage is influenced by operational parameters [17] as well as the relative motion of fiber tip over the tissue surface—factors critical for optimizing surgical outcomes in endourology [18]. For example, the highest stone ablation rates have been achieved with direct, orthogonal contact between the fiber tip and the stone surface [19, 20, 21].

Comparisons between pulsed lasers remain challenging due to differences in wavelength and pulse characteristics. The p‐Tm:YAG laser operates at 2013 nm, while TFL systems emit at 1908 or 1940 nm [22, 23], and Ho:YAG lasers at approximately 2100 nm [10, 24]. Water absorption coefficients thus differ by > 2‐fold at 37°C (Ho:YAG µ_a ≈ 2.5 mm^−1^; Tm:YAG ≈ 5.5–7 mm^−1^).

While first‐order Lambert‐Beer absorption (I ~ e^‐µa z^), scattering, and thermal diffusion govern energy deposition, endourology interactions prove more intricate: vapor bubble formation and cavitation are prominent with Ho:YAG pulses and dramatically alter tissue effects and stone ablation beyond linear predictions [22, 25, 26]. Different wavelengths yield distinct thermal relaxation times (τ_thermal_ ≈ 1/(4µ_a k); ~200–300 µs Ho:YAG vs. ~50–100 µs Tm:YAG), which create different thresholds for thermal confinement and mechanical disruption [27, 28]. These fundamental differences may drive the laser specific tissue effects seen experimentally and the varying safety profiles observed clinically [6, 28]. Further, peak power levels differ substantially (10.000 W for Ho:YAG vs. 3700 W for p‐Tm:YAG [29] and 500 W for TFL), and further distinctions such as pulse profiles [30, 31] may affect gas bubble dynamics, as demonstrated in high‐speed imaging studies [32]. These differences necessitate laser‐specific handling strategies, recently discussed in the context of enucleation [28, 33], and are equally critical in pulsed laser applications like lithotripsy.

This study aimed to compare soft tissue damage induced by the novel pulsed p‐Tm:YAG laser with that of Ho:YAG laser under standardized ex vivo conditions, in order to compare their procedural risks and safety margins in the context of RIRS.

## 2 Materials and Methods

Fresh porcine kidneys procured from a slaughterhouse were bisected and fixed in a pure water bath maintained at 37°C ± 0.5°C just prior to each experimental run to minimize potential osmotic effects (Eheim Thermocontrol 3604, Deizisau, Germany). Homogeneous parenchymal tissue without Gerota fascia and intact urothelial lining of the renal pelvis was positioned facing upward. The laser fiber was guided automatically along a linear trajectory by an XY‐actuator (Eleks, Lviv, Ukraine; Figure 1, left). The Z‐axis was adjusted to maintain a sub‐0.25 mm distance from the tissue surface ensuring near‐contact mode to minimize beam divergence effects while preventing fiber impaction and its subsequent adherence to bulged out tissue. Beam divergence from silica multimode fibers was reported with numerical aperture approximately 0.2–0.3, divergence half‐angles approximately 6° for both lasers, resulting in < 5% spot size variation over 250 µm with weak wavelength dependence [34]. Worst‐case tissue damage from complex laser‐tissue interaction was extracted by selecting from the large data pool (2.2).

**Figure 1:**
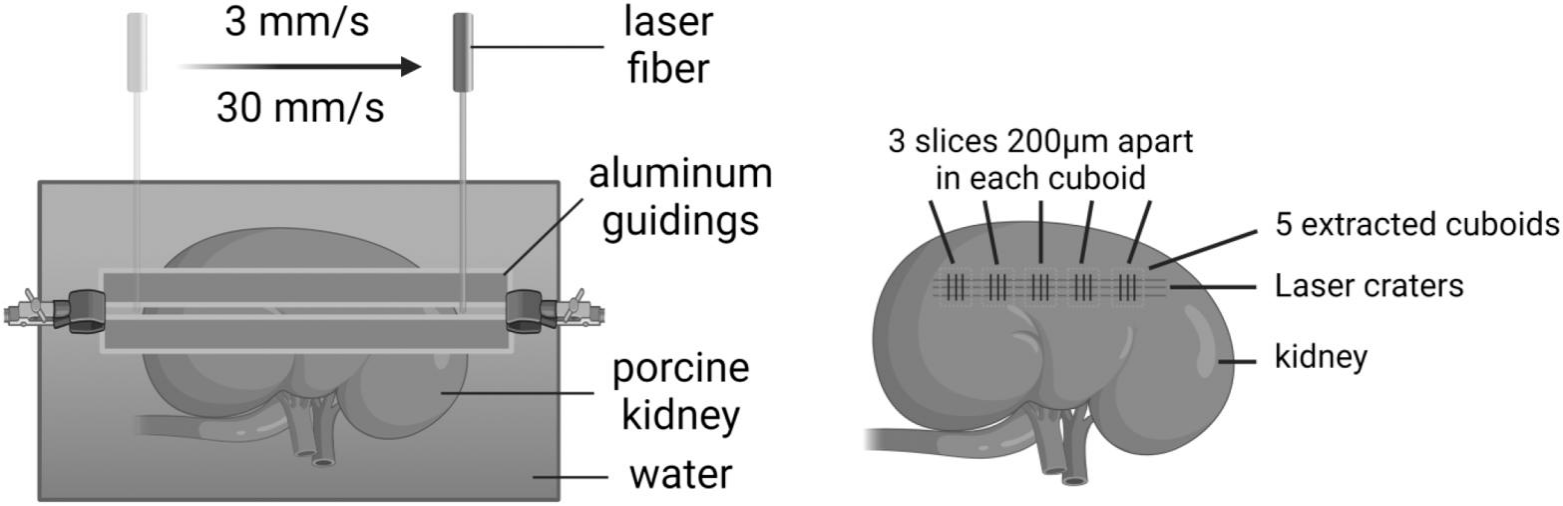
Experimental setup and histological sample extraction. Left: Top view of automated setup for horizontal motion of the fiber with constant distance to sample tissue. Right: 5 × 2–3 evaluable histological slices for each laser setting, 3 µm per slice.

### 2.1 Laser Devices and Parameters

A standard Ho:YAG device (Medilas H Solvo 35) was compared to a p‐Tm:YAG evaluation model (Thulioprecursor, Dornier MedTech Laser GmbH, Wessling, Germany). Laser fibers of 400 µm diameter were used in both lasers thus pulse energy and laser power scale equivalently to fluence (J/m^2^) and laser irradiance (W/m^2^).

The lasers were tested using parameter ranges comparable to lithotripsy (Supporting Information: Table 1). Pulse energy, frequency, and—where possible—pulse duration was matched across both systems at equivalent laser powers (Equation 1), serving as an efficiency marker for RIRS‐based lithotripsy. In addition, mean pulse power values were calculated (Equation 2).

(1) LaserPower=PulseEnergy×PulseFrequency

(2) $$\mathrm{Pulse}\;\mathrm{Power}=\frac{\mathrm{Pulse}\;\mathrm{Energy}}{\mathrm{Pulse}\;\mathrm{Duration}\;}$$

Experiments were conducted at a constant fiber velocity of 3 mm/s within comparable parameter ranges (Table 1). Additional tests using the p‐Tm:YAG device were performed at higher frequencies (100/200 Hz) and powers (60/80 W). Ho:YAG laser settings were limited to a maximum pulse duration of 450 µs and a power output of 35 W due to technical constraints (Supporting Information: Table 1).

**Table 1:** Margins of operational laser parameters for comparing tissue damage between the two technologies. A list of laser settings is accessible in Supporting Information: Table 1.

Laser type	Pulse energy [J]	Frequency [Hz]	Pulse length [µs]	Relative motion [mm/s]	Power^1^ [W]	Pulse power^2^ [W]
Ho:YAG	0.2–3.5	10–25	140–450	3	**2–35**	**1176–7777**
p‐Tm:YAG	0.2–3	10–40	150–1080	3	**2–45**	**950–3352**

Abbreviations: Ho:YAG, Holmium:Yttrium–Aluminum–Garnet; p‐Tm:YAG, pulsed‐Thulium: Yttrium–Aluminum–Garnet.

### 2.2 Histological and Statistical Analysis

After each experiment, the five most severely damaged tissue regions were excised into cuboids measuring 1–1.5 cm per edge (Figure 1, right) and fixed in formalin for a minimum of 5 days. Histological processing was performed at the Institute of Applied Photonics and Optics (Ulm, Germany), where three slices were cut from each cuboid at 200 µm intervals, yielding 10–22 samples per parameter set. The slices were dehydrated at 37°C for 1 h using a SLEE MTMII system under pressure and vacuum, then embedded in paraffin using ascending ethanol/xylene concentrations (Paraplast Kendall 2502004).

Crater depth and axial/lateral coagulation thickness were defined as quantitative endpoints for histological analysis (Bresser, Rhede, Germany; Figure 3). These parameters were initially assessed in relation to laser power and pulse power. Within clinically relevant corridors—defined by equivalent pulse energies and comparable laser powers (Supporting Information: Table 1)—3D tissue‐specific point clouds of histological endpoints were generated. An unsupervised k‐means clustering approach based on nearest‐neighbor distances was used to differentiate damage profiles predominantly associated with Ho:YAG and p‐Tm:YAG. To avoid convergence to local minima, clustering was repeated 25 times, and the most robust solution was selected. A subsequent subgroup analysis examined the associated laser parameters of each cluster. Additional damage caused by the p‐Tm:YAG laser at high power (60–80 W) or high frequency (up to 200 Hz) was analyzed separately for renal parenchyma using the Wilcoxon test.

## 3 Results

### 3.1 Laser Parameters

Direct impact scenarios of Ho:YAG and p‐Tm:YAG laser beams were compared using realistic scenarios of laser parameter and comparable laser powers (Table 1). The resulting single‐pulse power for p‐Tm:YAG peaked at 3362 W versus for Ho:YAG at 7777 W (Equation 2). Even explorations with p‐Tm:YAG at higher laser powers (max. 80 W) did not exceed its pulse power limit of 3362 W (Supporting Information: Table 1).

### 3.2 Damage Analyses

Shallow and deep damage patterns with cuts and coagulation zones were visible macroscopically on renal parenchyma and pelvis (Figure 2) with visibly deeper cuts at higher power Ho:YAG laser applications. Confluent single craters were visible on renal parenchyma (Figure 2A–C) and renal pelvis (Figure 2D), which often merged into trajectories of craters. Both renal tissues showed a well definable coagulation lining (Figure 2A renal pelvis, 2B–D parenchyma).

**Figure 2:**
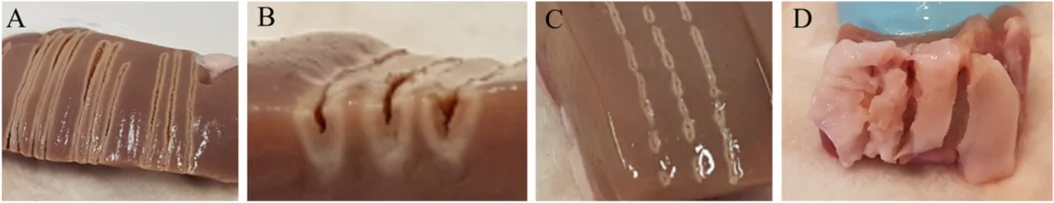
Macroscopic damage on renal parenchyma (A–C) and renal pelvis (D).

A variety of injury profiles were histologically documented and quantified by cutting depth and axial and lateral coagulation thickness (Figure 3; 560 histological slices). For Ho:YAG, delamination and tissue detachments were prominent at the crater surface (Figure 4, left column). For p‐Tm:YAG, coagulation zones appeared denser and compact (Figure 4, right columns). In the renal pelvis, more pronounced coagulation zones and shallow craters with an arching up of surrounding fibers were typical.

**Figure 3:**
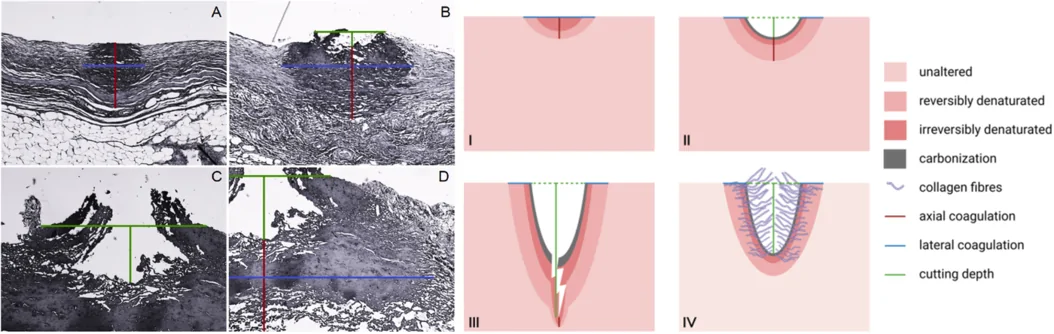
Quantification of damage from histological analyses, vertical green line as reference level (A/I) coagulation only, (B/II) minimal cutting depth, (C/IV) cutting through collagenous fibers, (III) ripped crater, (D) broad lateral coagulation around fibrous crater. Red/blue: axial/lateral coagulation, green: cutting depth.

**Figure 4:**
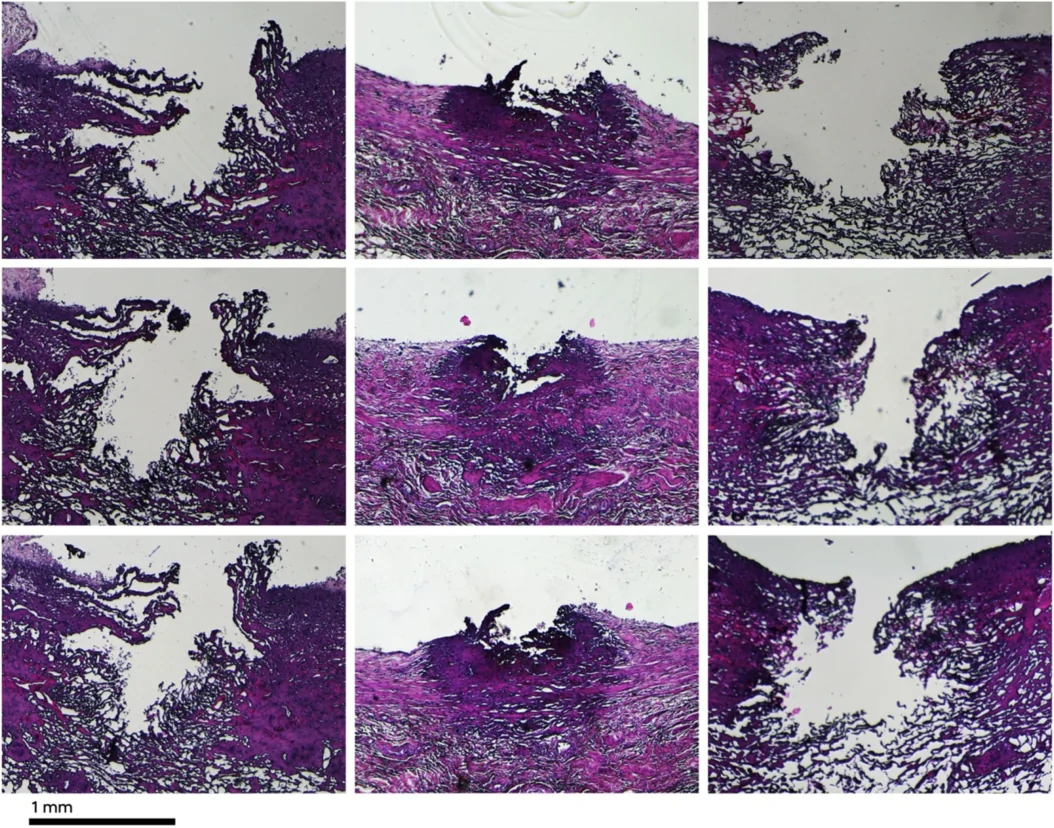
Histological differences in cutting and coagulation behavior on renal pelvis; left: Ho:YAG, 10 Hz, 220 µs, 0.6 J, 6 W; middle: p‐Tm:YAG, 10 Hz, 192 µs, 0.6 J, 6 W; right: p‐Tm:YAG, 15 Hz, 842 µs, 0.8 J, 12 W. Ho:YAG, Holmium:Yttrium–Aluminum–Garnet; p‐Tm:YAG, pulsed‐Thulium: Yttrium–Aluminum–Garnet

Ho:YAG cut between 0.6 and 9.3 mm within renal parenchyma, while p‐Tm:YAG cut between 0.3 and 5.0 mm (Table 2). In the renal pelvis, maximum cut depths were lower, reaching 3.6 mm with Ho:YAG but also nearly doubled compared to p‐Tm:YAG (2.0 mm). One p‐Tm:YAG trajectory resulted in no cutting damage across all cross sections, employing low power and low pulse power (2 W/1176 W; Supporting Information: Table 2). Given equal laser parameters, mean coagulation thickness was a 1.4–4.5‐fold in renal pelvis. However, also variations in coagulation thickness doubled.

**Table 2:** Descriptive overview of damage from within the comparable parameter corridor. *n* = 10–15 slices per parameter set (Supporting Information: Table 2,) with fixed laser kinematics at 3 mm/s, fixed distance, orthogonal direction.

	Renal parenchyma		Renal pelvis	
	Ho:YAG *n* = 110	p‐Tm:YAG *n* = 135	Ho:YAG *n* = 97	p‐Tm:YAG *n* = 101
Cutting depth [mm]	3.05 (0.60–9.30)	2.81 (1.10–5.00)	1.38 (0.05–3.60)	0.61 (0.00–2.00)
Axial coagulation depth [mm]	0.16 (0.10–0.70)	0.34 (0.10–0.75)	0.73 (0.05–1.80)	0.62 (0.30–1.20)
Lateral coagulation depth [mm]	0.49 (0.00–0.90)	0.57 (0.15–0.85)	1.02 (0.10–1.60)	0.79 (0.20–1.40)

*Note:* Mean (Min–Max).

Abbreviations: Ho:YAG, Holmium:Yttrium–Aluminum–Garnet; p‐Tm:YAG, pulsed‐Thulium: Yttrium–Aluminum–Garnet.

### 3.3 Associations With Laser Parameters

Cutting depths correlated with both laser power and pulse power (Figure 5). In renal parenchyma, Ho:YAG laser cut an average of 0.177 mm/W (0.116 mm/W in the renal pelvis), p‐Tm:YAG cut at reduced slopes of 0.050 mm/W in parenchyma (0.038 mm/W in the pelvis; left). The variance in cut depths was less for renal parenchyma and cut depths was generally better explained for Ho:YAG (*R* = 0.82), while cut depths with p‐Tm:YAG was less linear (*R* = 0.28).

**Figure 5:**
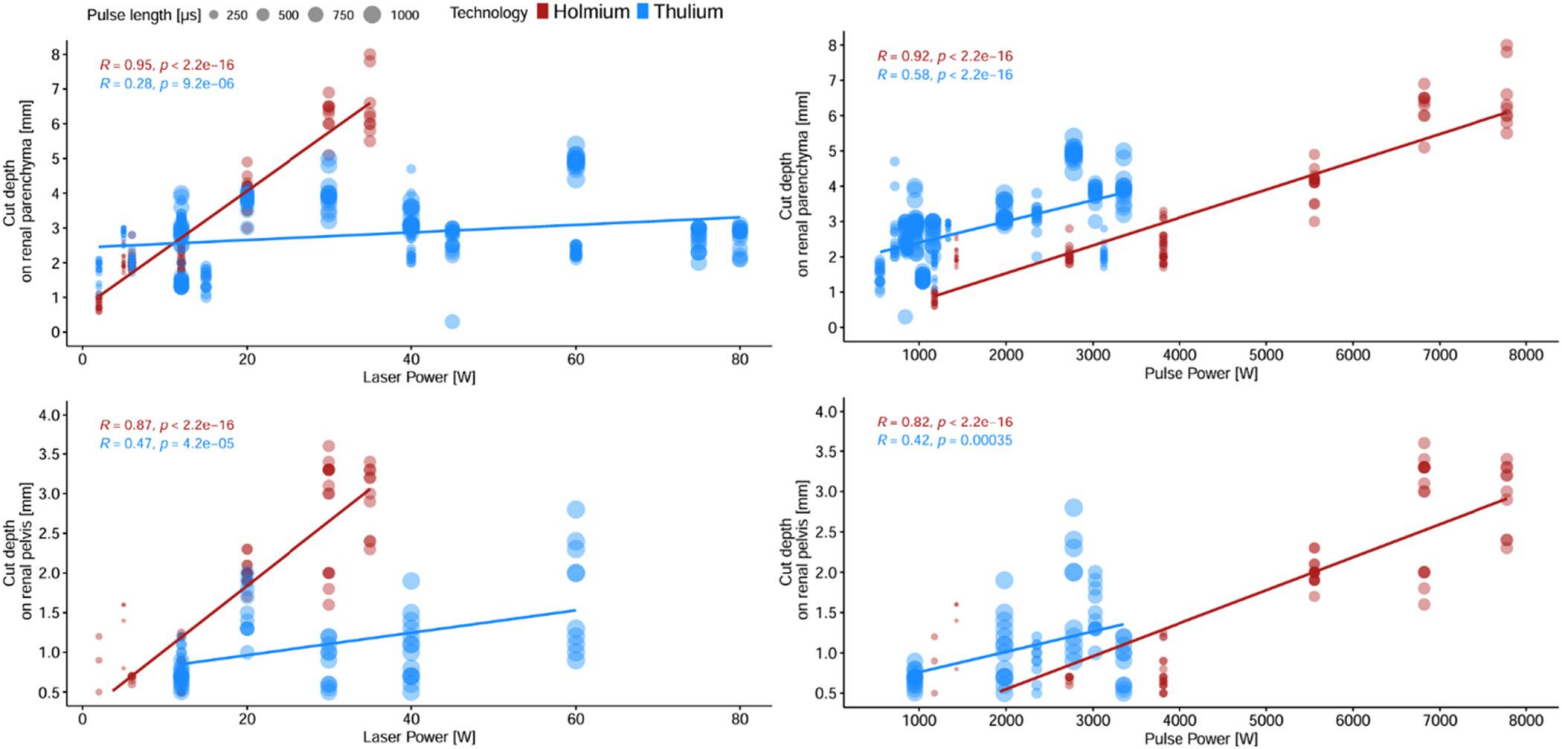
Cut depths displayed along the laser power (Equation 1, left) and its pulse power (Equation 2, right). Each point represents the cut depth from one histological slide from repeated measurements of cut depths. Marker size indicates the employed pulse lengths.

As a result, the differences between the two laser systems became most pronounced at higher power levels. Even in the extended power range up to 80 W—achievable only with p‐Tm:YAG—cut depths remained below those observed with Ho:YAG (Figure 5).

P‐Tm:YAG natively emits longer pulses and as a consequence reduced pulse power (right/Table 2). This concept partly explained the observed technology differences, as the correlation with pulse power was less drastic, at similar coefficients.

Applying p‐Tm:YAG at high pulse frequencies (75/100/200 Hz; *n* = 95) did not result in deeper cuts in renal parenchyma but rather led to enhanced coagulation (*p* < 0.001).

Axial coagulation with the p‐Tm:YAG was approximately twice that observed with the Ho:YAG in renal parenchyma. In renal pelvis, variability of coagulation measurements was higher and correlations with either power or single‐pulse power were unspecific.

### 3.4 Associations with Laser Technologies

Based on cut depth, axial, and lateral coagulation, distinguished tissue impact profiles were generated by an unsupervised nearest‐neighbour approach on damage data from the comparable power corridor 2–40 W. In renal parenchyma, this clustering yielded four distinct application or risk regimes (cluster sizes: 21–82 measurements; Figure 6)

- **Cluster 1** comprised a mix of both laser types (47 p‐Tm:YAG/34 Ho:YAG) and was characterized by minimal lateral coagulation (< 0.4 mm) and shallow cut depths (< 2 mm), resulting from low pulse energies and minimal laser powers below 10 W.
- **Cluster 2**, also evenly mixed (42 p‐Tm:YAG/40 Ho:YAG), showed moderate cut depths (< 3.5 mm) and average coagulation (axial: < 0.2 mm; lateral: 0.6 mm). These profiles arose from medium pulse power settings—high power and long pulses for p‐Tm:YAG, and medium power with shorter pulses for Ho:YAG.
- **Cluster 3** consisted predominantly of p‐Tm:YAG measurements (58/61; 95%) and displayed moderate cut depths (< 4 mm) with pronounced axial coagulation (> 0.5 mm), more than twice the levels observed in other clusters. This damage pattern was associated with high pulse durations and energy levels, resulting in high average power (up to 40 W) but relatively low pulse power below 4000 W.
- **Cluster 4** was largely Ho:YAG‐derived (20/21; 95%) and exhibited the most aggressive damage profile, with the deepest cuts (up to 9.3 mm) and low axial and lateral coagulation (< 0.5 mm). These were linked to high pulse energy, average power (> 20 W) and highest pulse power levels 6000–8000 W.

**Figure 6:**
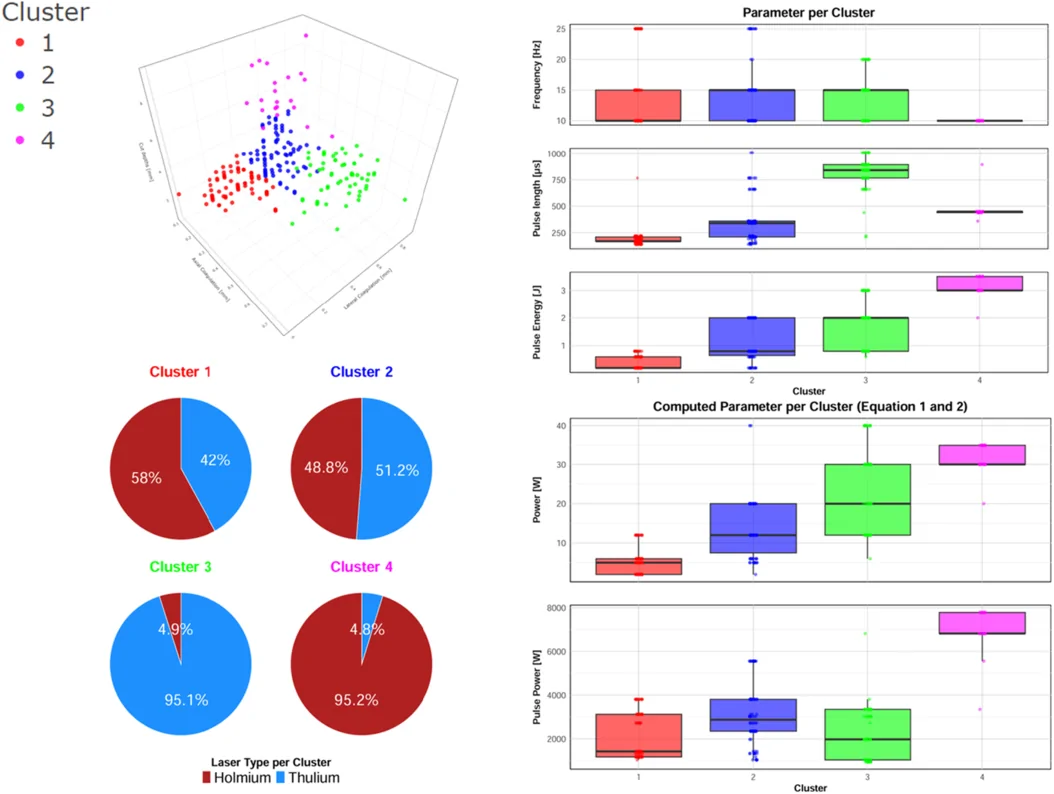
Nearest neighbors clustering of the injury measures on renal parenchyma divided into one Ho:YAG, one p‐Tm:YAG and two mixed clusters (left). Laser parameters from within these clusters show characteristic margins (interquartile range and 95% confidence interval [CI], right). Ho:YAG, Holmium:Yttrium–Aluminum–Garnet; p‐Tm:YAG, pulsed‐Thulium: Yttrium–Aluminum–Garnet.

In renal pelvis tissue, clustering was less effective in distinguishing p‐Tm:YAG from Ho:YAG in the low to medium laser power regime (Supporting Information: Figure 1). Among four clusters, only the high power and high pulse power cluster (> 20 W/ > 6000 W) stood out by representing more than 75% Ho:YAG measurements. They corresponded to the deepest cuts (> 2 mm) and moderate axial coagulation around 1 mm. The remaining clusters were mixed and predominantly composed of p‐Tm:YAG cases. The distinctive coagulation behavior that separated p‐Tm:YAG damage profiles in the parenchyma was not observed in the structurally heterogeneous renal pelvis. A separate cluster comprising ≥ 75% p‐Tm:YAG cases would have required increasing the number of clusters to seven, which was not pursued.

## 4 Discussion

Both environment pulsed solid‐state Ho:YAG and p‐Tm:YAG lasers have been investigated for their tissue effects on porcine renal tissues over a variety of laser parameters in the context of RIRS. The results demonstrate that laser parameters are the primary determinants of tissue injury. Elevated laser power presents the highest risk, however, due to the differing physical properties of Ho:YAG and p‐Tm:YAG lasers i.e. their differing wavelengths, pulse parameter selection is inherently constrained, and the resulting damage profiles are closely tied to the specific laser technology used.

### 4.1 Cutting Depths

For Ho:YAG and p‐Tm:YAG, damage depth increases with laser power, but the rate of increase is reduced for longer‐pulsed p‐Tm:YAG lasers showing lower cut depths persistent to high powers of 50 to 80 W. While this comparative investigation is novel for pulsed laser systems for RIRS, findings relate to histological studies comparing soft tissue effects in the context of ablation and enucleation. A recent study on enucleation of the prostate compared Ho:YAG and continuous wave Thulium laser (cw‐Tm:YAG), continuous laser emission was concluded beneficial for precise dissection and safe coagulation [35]. In the context of RIRS, the extent of cut damage presented ex‐vivo at realistic but most severe conditions for renal tissue may be causal to complications. The standard, short‐pulsed characteristic of Ho:YAG has earlier been linked to safety concerns [36, 37] and correlations between laser power and thermal damage have been established experimentally [38]. This study motivates further studies on pulse power and temporal energy peaks of novel laser systems, and to limit applied pulse power rather than total laser power to reduce complications during RIRS. More studies are needed to transfer these concepts to efficiency analyses of stone ablation, fragmentation and dusting.

### 4.2 Coagulation

Damage profiles from p‐Tm:YAG and Ho:YAG vary due to their individual coagulation aspects, with p‐Tm:YAG showing more extensive and qualitatively more dense and compact coagulation zones (Figure 4, Cluster 1 and 3, Figure 6). These were persistent to high powers beyond 50 W. A beneficial coagulation for this technology is supported by experimental findings on pulsed Tm:YAG, showing denser coagulation zones without carbonization when compared to Ho:YAG [28]. The histological presentation may explain high safety levels of this novel device in clinical use [39]. Interestingly, these results are also consistent for continuous wave lasers compared to Ho:YAG, which more often necessitates secondary coagulation [35, 38, 40]. However, differences on cut depths and coagulation had not been identified in an earlier study with fewer experiments [40], although both studies characterized histological damage profiles. This discrepancy indicates that differences are not extensive for all laser parameters and may for some application i.e. with low power be non‐existent or small. Automation and high sample size as well as homogenous tissue samples can simplify their detection.

### 4.3 Laser Parameters

Despite high available power input necessary for effective stone ablation, fragmentation and dusting [17, 20, 29], longer pulses native to p‐Tm YAG may be a feature resulting in the less invasive tissue damage. This hypotheses had already been suggested in the context of cw‐Tm:YAG [27], and extends the previous view on factors for damage during lithotripsy to pulse parameters [41].

Pulse duration—or the combined parameter of pulse power level (Equation 2)—was identified as a decisive additional factor with thermal and biomechanical consequences on tissue. For Ho:YAG, thermal confinement criterion (τ_thermal_ = 286 ms [42]) was met or only slightly exceeded by pulse duration (140–450 ms). For p‐Tm:YAG (assuming in comparable order as TFL τ_thermal_ = 11.4 ms [43]), all the typically applied pulses are a 10‐100fold longer than necessary for damage to stay locally confined (pulse duration 150–1080 ms). This may explain the increase and more reliable thermal coagulation observed for p‐Tm:YAG, and the mechanical damage from confined energy absorption from Ho:YAG. Besides the benefits discussed here, there are also associated risk for unspecific heat generation which had been recently discussed [43], motivating more research on thermal safety in endourological laser development.

It is suggested that pulse duration should gain more attention clinically and in further clinical studies, that is, by comparing safety and efficiency aspects regarding pulse energy profiles. For Tm:YAG, rectangular pulses had been documented [44], following the proposed mean pulse power of Equation (2). On the contrary, Ho:YAG exhibits asymmetrical pulse shapes, with a steep leading slope and a much more gradual trailing slope [30, 31], adding to the already spike‐like character of short Ho:YAG pulses.

High frequencies (75–200 Hz) with p‐Tm:YAG yielded no deeper parenchymal cuts but rather increased coagulation zones (*p* < 0.001), attributable to more homogenous power intake with overlapping thermal zones, favoring coagulation over vaporization and ablation [42]. Elevated pulse frequencies comprise a valuable parameter for improving effectiveness of RIRS, that is, for dusting [17, 20, 29]. While this is the first study demonstrating tissue safety as a function of pulsed parameter margins for p‐Tm:YAG, for Ho:YAG low‐energy and high‐frequency pulses had previously been documented as superior in terms of safety and efficiency in an experimental setup [6], as well as in their clinical outcome [45]. In view of the general risk associated with high laser powers, the presented results suggest high frequency options to be a safe measure, and it would be interesting to see these options being further developed in future devices. However, high frequencies may add up other procedural risks such as cumulative heat buildup and challenges with irrigation and cooling capacity [46]. Moreover, multiple impacts at a single location may have serious consequences [11], as also highlighted by the extensive damage to the renal parenchyma observed in this study.

### 4.4 Limitations

This study has several limitations. Most notably, it was conducted under ex vivo laboratory conditions, which do not fully replicate the dynamic environment of the human upper urinary tract during RIRS. Factors such as tissue perfusion, bleeding, and irrigation—each of which may influence laser–tissue interactions in vivo—were not addressed. In addition, osmotic reactions of renal tissue may have influenced the results, although procedural times were kept within minutes.

In this study, the distance between the fiber tip and the curved, compliant tissue surface was maintained below 250 µm, which represented the best achievable consistency for the pulsed laser system, but still constitutes a source of measurement variability. The vertical distance of the fiber tip influences energy delivery to the tissue. Photothermal models and laser beam divergence predict decreased energy deposition with increasing distance [42]. However, laser–tissue interactions in endourological applications are governed by phenomena such as vapor bubble dynamics, plasma formation, and scattering, making energy delivery and tissue damage difficult to predict and not strictly decreasing with increasing fiber–tissue distance [26]. Multiple trajectories were generated for each parameter set, and histological analysis focused on samples exhibiting the greatest depth of damage to capture worst‐case scenarios. Both lasers were applied at identical conditions, but the larger diffusion for Thulium could make the distance a relevant value, potentially underestimating tissue damage.

Furthermore, the study focused on comparing Ho:YAG and p‐Tm:YAG lasers on renal model tissues, and conclusions cannot be generalized to all thulium‐based laser systems, that is, TFL and other, more inhomogeneous tissues may interact with larger variations. However, studies on ureteral damage referred to above do not contradict the presented evidence.

## 5 Conclusion

Any unintended emission of laser energy onto tissue represents a medical risk, which is, beyond procedural and kinetic factors, mainly based on the laser type and settings. High‐power Ho:YAG is associated with greatest complication risk due to deeper injuries and less pronounced protective coagulation zones.

Tantamount to an automated setup with a homogenous model tissue and multidimensional sweeps of laser parameters, individual safety zones were identified including high powers beyond 50 W and high frequencies to 200 Hz. High energy impacts from Ho:YAG lasers are more likely to produce deeper craters with reduced and less compact coagulation, potentially resulting in procedural or clinical complications if not immediately coagulated or sealed.

Pulse power was a preliminary determinant of cut depth for both technologies. Inherently reduced pulse powers of p‐Tm:YAG may be a safety benefit during high power applications such as RIRS. Additionally, protective coagulation is more likely to be achieved with longer pulses Ho:YAG and are characteristics of p‐Tm:YAG impacts, persistent to high power levels. Milder damage depths, superior coagulation profiles persistent to high powers and stable depths at high frequencies makes p‐Tm:YAG a promising candidate for highly effective RIRS lithotripsy.

## Conflicts of Interest

No competing financial interests exist. Dennis Buente has nothing to disclose. Rodrigo Suarez‐Ibarrola has nothing to disclose. Arkadiusz Miernik receives research funding from the German Federal Ministry of Education and Research, Berlin (D). He receives support for his travel activities from the European Association of Urology, Arnhem (NL), and German Society of Urology, Düsseldorf (D). Arkadiusz Miernik is also a consultant for: KLS Martin, Tuttlingen (D), Dornier MedTech Laser GmbH (D), Optimed (D), Richard Wolf GmbH (D), Boston Scientific (USA), Gimmi (D) and Filax medical (CH). Shane O'Sullivan has nothing to disclose. Ralf Petzold was consultants to Dornier MedTech Laser GmbH (D) at the time of the measurements. The team received an evaluation p‐Tm:YAG device for ex‐vivo testing.

## Supporting information

Supporting File

## Data Availability

The data that support the findings of this study are available from the corresponding author upon reasonable request.
