# Supplementary material for: Soft Tissue Safety Assessment of Pulsed Solid‐State Thulium:YAG Versus Holmium:YAG Lasers: A Quantitative Porcine Model Simulation
Source: Lasers Surg Med. 2026 Jul 15;58(9):811–20. doi: 10.1002/lsm.70173 (PMC13649090; doi:10.1002/lsm.70173)
Supplement: Supplementary file 1 [file LSM-58-811-s001.docx]

# Supplementary Material

Suppl. Fig. 1 Unsupervised learning on damage outcomes on renal pelvis. The outcome measures were divided into one 75% Ho:YAG cluster (20/7), the other clusters were mixed (left). Laser parameters from within these clusters are shown on the right.

Suppl. Tab. 1 Parameter sets. designed to compare by mean laser power. Extensions to high powers and high velocity damage for p-Tm:YAG

| **Tissue and Laser** | Pulse duration [µs] | Pulse energy [J] | Frequency [Hz] | Power [W] | Pulse Power [W] |
| --- | --- | --- | --- | --- | --- |
| **Ho:YAG**  **on renal pelvis** | 170 | 0,2 | 10 | 2 | 1176 |
|  | 140 | 0,2 | 25 | 5 | 1429 |
|  | 220 | 0,6 | 10 | 6 | 2727 |
|  | 210 | 0,8 | 15 | 12 | 3810 |
|  | 360 | 2 | 10 | 20 | 5556 |
|  | 440 | 3 | 10 | 30 | 6818 |
|  | 450 | 3,5 | 10 | 35 | 7778 |
| **Ho:YAG**  **on renal parenchym** | 170 | 0,2 | 10 | 2 | 1176 |
|  | 140 | 0,2 | 25 | 5 | 1429 |
|  | 220 | 0,6 | 10 | 6 | 2727 |
|  | 210 | 0,8 | 15 | 12 | 3810 |
|  | 360 | 2 | 10 | 20 | 5556 |
|  | 440 | 3 | 10 | 30 | 6818 |
|  | 450 | 3,5 | 10 | 35 | 7778 |
| **p-Tm:YAG**  **on renal pelvis** | 170 | 0,2 | 10 | 2 | 1176 |
|  | 140 | 0,2 | 25 | 5 | 1429 |
|  | 220 | 0,6 | 10 | 6 | 2727 |
|  | 210 | 0,8 | 15 | 12 | 3810 |
|  | 360 | 2 | 10 | 20 | 5556 |
|  | 440 | 3 | 10 | 30 | 6818 |
|  | 450 | 3,5 | 10 | 35 | 7778 |
| **p-Tm:YAG**  **on renal parenchym** | 170 | 0,2 | 10 | 2 | 1176 |
|  | 150 | 0,2 | 25 | 5 | 1333 |
|  | 192 | 0,6 | 10 | 6 | 3125 |
|  | 842 | 0,8 | 15 | 12 | 950 |
|  | 768 | 0,8 | 15 | 12 | 1042 |
|  | 368 | 0,2 | 75 | 15 | 543 |
|  | 661 | 2 | 10 | 20 | 3026 |
|  | 895 | 3 | 10 | 30 | 3352 |
|  | 1010 | 2 | 20 | 40 | 1980 |
|  | 278 | 0,2 | 200 | 40 | 719 |
|  | 714 | 0,6 | 75 | 45 | 840 |
|  | 1080 | 3 | 20 | 60 | 2778 |
|  | 464 | 0,4 | 150 | 60 | 862 |
|  | 864 | 1 | 75 | 75 | 1157 |
|  | 828 | 0,8 | 100 | 80 | 966 |

Suppl. Tab. 2 Descriptive Data from histological slices (n=10-22)

| **Tissue** | **Mean laser Power**  **[W]** | **Pulse duration**  **Ho:YAG / p-Tm:YAG** | Ho:YAG Cutting depth [mm] | | | p-Tm:YAG Cutting depth [mm] | | | Ho:YAG axial Coagulation [mm] | | | p-Tm:YAG axial Coagulation [mm] | | | Ho:YAG lateral Coagulation [mm] | | | p-Tm:YAG lateral Coagulation [mm] | | |
| --- | --- | --- | --- | --- | --- | --- | --- | --- | --- | --- | --- | --- | --- | --- | --- | --- | --- | --- | --- | --- |
| **pelvis** | 2 | 170/170 | **0.43** | ± | 0.37 | **0.00** | ± | 0.00 | **0.42** | ± | 0.38 | **0.48** | ± | 0.14 | **0.53** | ± | 0.29 | **0.42** | ± | 0.38 |
|  | 5 | 140/150 | **0.64** | ± | 0.66 | **0.20** | ± | 0.14 | **0.44** | ± | 0.35 | **0.75** | ± | 0.08 | **0.72** | ± | 0.09 | **0.44** | ± | 0.35 |
|  | 6 | 220/192 | **0.49** | ± | 0.25 | **0.12** | ± | 0.09 | **0.37** | ± | 0.24 | **0.5** | ± | 0.10 | **0.54** | ± | 0.21 | **0.37** | ± | 0.24 |
|  | 12 | 210/340  &-/842 | **0.65** | ± | 0.23 | **0.91**  **0.69** | ±  ± | 0.22  0.10 | **0.55** | ± | 0.11 | **0.55**  **0.46** | ± | 0.10  0.09 | **0.55** | ± | 0.17 | **0.99**  **0.15** |  | 0.15  0.26 |
|  | 20 | 360/661 | **2.01** | ± | 0.15 | **1.52** | ± | 0.32 | **0.98** | ± | 0.12 | **1** | ± | 0.23 | **1.12** | ± | 0.24 |  |  |  |
|  | 30 | 440/895 | **2.80** | ± | 0.70 | **0.87** | ± | 0.28 | **1.07** | ± | 0.12 | **0.64** | ± | 0.08 | **1.11** | ± | 0.13 | **1** | ± | 0.16 |
|  | 35 | 450/ - | **2.94** | ± | 0.42 |  |  |  | **1.22** | ± | 0.28 |  |  |  | **1.39** |  | 0.17 |  |  |  |
|  | 40 | -/1010 |  |  |  | **0.99** | ± | 0.42 |  |  | 0.14 | **0.66** | ± | 0.18 |  | ± |  | **1.02** |  | 0.19 |
|  | 60 | *-/1080* | **0.49** | ± | 0.15 | **1.70** | ± | 0.68 |  | ± | 0.12 | ***0.72*** |  | *0.16* |  |  |  | **1.04** |  | 0.23 |
| **parenchym** | **Mean laser Power**  **[W]** | **Pulse duration**  **Ho/Tm** | Ho Cutting depth [mm] | | | Tm Cutting depth [mm] | | | Ho axial Coagulation [mm] | | | Tm axial Coagulation [mm] | | | Ho lateral Coagulation [mm] | | | Tm lateral Coagulation [mm] | | |
|  | 2 | 170/170 | **0.83** | ± | 0.16 | 1.79 | ± |  | **0.12** | ± | 0.03 |  | ± |  | **0.21** | ± | 0.04 |  | ± |  |
|  | 5 | 140/150 | **2.04** | ± | 0.25 | 2.85 | ± |  | **0.12** | ± | 0.04 |  | ± |  | **0.35** | ± | 0.10 |  | ± |  |
|  | 6 | 220/192 | **2.05** | ± | 0.25 | 2.07 | ± |  | **0.26** | ± | 0.11 |  | ± |  | **0.42** | ± | 0.08 |  | ± |  |
|  | 12 | 210/768  -/842 | **2.28** |  | 0.40 | 1.40  3.03 | ±  ± | 0.10  0.45 | **0.17** |  | 0.09 | 0.38  0.55 | ±  ± | 0.10  0.06 | **0.53** | ± | 0.13 | 0.60  0.71 | ± | 0.09  0.05 |
|  | 15 | -/368 |  |  |  | 1.50 | ± | 0.27 |  |  |  | 0.53 | ± | 0.06 |  |  |  | 0.63 | ± | 0.08 |
|  | 20 | 360/661 | **4.07** | ± | 0.45 | 3.79 | ± | 0.27 | **0.11** | ± | 0.03 | 0.33 | ± | 0.11 | **0.68** | ± | 0.07 | 0.64 | ± | 0.06 |
|  | 30 | 440/895 | **6.55** | ± | 1.08 | 3.95 | ± | 0.47 | **0.21** | ± | 0.18 | 0.85 | ± | 1.29 | **0.70** | ± | 0.12 | 0.75 | ± | 0.05 |
|  | 35 | 450/- | **6.63** | ± | 0.97 |  |  |  | **0.19** | ± | 0.13 |  |  |  | **0.59** | ± | 0.06 |  | ± |  |
|  | 40 | -/278  -/1010 |  |  |  | 2.623.25 | ± | 0.770.29 |  | ± |  | 0.450.42 | ± | 0.160.09 |  |  |  | 0.80  0.74 | ± | 0.16  0.09 |
|  | 45 | -/714 |  |  |  | 2.74 | ± | 0.28 |  | ± |  | 0.45 | ± | 0.12 |  |  |  | 0.69 | ± | 0.09 |
|  | 60 | -/464  -/1080 |  |  |  | 2.324.92 | ± | 0.140.22 |  | ± |  | 0.790.42 | ± | 0.090.15 |  |  |  | 0.96  0.85 | ± | 0.12 0.14 |
|  | 75 | 864 |  |  |  | 2.68 | ± | 0.33 |  | ± |  | 0.60 | ± | 0.14 |  |  |  | 0.94 | ± | 0.08 |
|  | 80 | 828 |  |  |  | 2.73 | ± | 0.35 |  | ± |  | 0.52 | ± | 0.15 |  |  |  | 1.06 | ± | 0.12 |
